# Synthesis and In Vitro Characterization of Glycopeptide Drug Candidates Related to PACAP_1–23_

**DOI:** 10.3390/molecules26164932

**Published:** 2021-08-14

**Authors:** Christopher R. Apostol, Parthasaradhireddy Tanguturi, Lajos Z. Szabò, Daniel Varela, Thiago Gilmartin, John M. Streicher, Robin Polt

**Affiliations:** 1 Department of Chemistry and Biochemistry, BIO5, The University of Arizona, 1306 E. University Blvd, Tucson, AZ 85721, USA; caposto1@email.arizona.edu (C.R.A.); lzszabo@arizona.edu (L.Z.S.); 2 Department of Pharmacology, College of Medicine, The University of Arizona, 1501 N. Campbell Ave, Tucson, AZ 85724, USA; parthasaradhit@arizona.edu (P.T.); jstreicher@arizona.edu (J.M.S.); 3 Facultat de Quìmica Tarragona, Universitat Rovera I Virgili, 43007 Barcelona, Spain; danielvarela@email.arizona.edu (D.V.); thiagogilmartin@email.arizona.edu (T.G.)

**Keywords:** glycosylation, glycopeptide, stroke, Alzheimer’s, Parkinson’s

## Abstract

The search for efficacious treatment of neurodegenerative and progressive neuroinflammatory diseases continues, as current therapies are unable to halt or reverse disease progression. PACAP represents one potential therapeutic that provides neuroprotection effects on neurons, and also modulates inflammatory responses and circulation within the brain. However, PACAP is a relatively long peptide hormone that is not trivial to synthesize. Based on previous observations that the shortened isoform PACAP_1–23_ is capable of inducing neuroprotection in vitro, we were inspired to synthesize shortened glycopeptide analogues of PACAP_1–23_. Herein, we report the synthesis and in vitro characterization of glycosylated PACAP_1–23_ analogues that interact strongly with the PAC1 and VPAC1 receptors, while showing reduced activity at the VPAC2 receptor.

## 1. Introduction

Neurodegenerative disorders continue to negatively impact the health and quality of life of millions of people worldwide [1,2,3,4]. Many current therapeutic strategies involve only symptomatic alleviation, as there is a severe lack of treatments with the potential to halt or reverse disease progression [5,6]. A plethora of studies have elucidated the molecular pathophysiology of various neurodegenerative diseases, revealing a complex interplay between neuronal apoptosis (oxidative stress, mitochondrial dysfunction, imbalances in ion homeostasis) and the inflammatory response in the brain [7,8,9,10,11]. Hyperactivation of microglia, the resident macrophages of the brain, results in the continual release of pro-inflammatory cytokines, which induces oxidative stress, mitochondrial dysfunction, imbalances in ion homeostasis, and eventual cell death. These apoptotic events will induce further stimulation of hyperactive microglia, establishing a continual cycle of neuronal cell death and neuroinflammation that promotes disease progression [7,12,13]. Thus, one of the biggest challenges associated with treating neurodegenerative diseases is effectively addressing both the apoptotic and neuroinflammatory aspects simultaneously. Endogenous pleiotropic peptide hormones are a representative class of compounds that may be able to address this critical problem. The pituitary adenylate cyclase activating peptide (PACAP) is one such endogenous peptide that has been shown to elicit neuroprotection and anti-inflammatory activity in animal models of Parkinson’s disease (PD), ischemic stroke, Alzheimer’s disease (AD), traumatic brain injury (TBI), and ethanol toxicity [14,15,16,17,18,19,20,21]. Interestingly, PACAP’s primary sequence has been conserved for millions of years across different species, which implies that it regulates critical biological functions [22]. The wide distribution of PACAP and its cognate receptors in different organ systems throughout the body further indicates their important regulatory roles [23]. PACAP exists as two different isoforms containing either 27 or 38 amino acid residues (Table 1). PACAP’s biological activities are elicited through three class B G-protein coupled receptors (GPCRs) known as PAC1, VPAC1, and VPAC2. Of note, class B GPCRs contain a large extracellular domain thought to be an affinity trap to initially bind their relatively large cognate peptide ligands, making them structurally distinct from other members of the GPCR family tree [24,25]. PACAP’s neuroprotective effects are mediated through PAC1, while immunomodulatory effects are modulated through VPAC1 and VPAC2 [23]. More specifically, VPAC1 activation elicits the production of anti-inflammatory mediators, while activation of VPAC2 can induce a pro-inflammatory response. Thus, relatively lower affinity for VPAC2 is desirable. PACAP binds and activates PAC1, VPAC1, and VPAC2 with equally high affinity whereas the vasoactive intestinal peptide (VIP), a structurally homologous relative of PACAP (Table 1), exhibits high affinity for VPAC1 and VPAC2, but is not selective for PAC1. Due to the promiscuous binding profile of PACAP, several research groups have attempted to prepare PAC1-selective PACAP analogues with potent neuroprotective activity. Some success has been attained, but there is yet to be a synthetic purely PAC1-selective agonist with no activity at the VPAC1/VPAC2 receptors [26]. This difference in affinity is intriguing considering there is striking structural similarity between VIP and PACAP_1–27_ (Table 1). However, positions 4 and 5 in PACAP’s and VIP’s primary sequences may be the key to fine-tuning receptor selectivity [27,28]. Positions 4 and 5 in PACAP are occupied by Gly and Ile, respectively, whereas the corresponding residues in VIP are Ala and Val. Gly is a known β-turn inducer while Ala is known to promote α-helical conformations, and Ile and Val have subtly different steric profiles. Thus, it is hypothesized that amino acid substitutions in the “hinge region” may lead to analogues with more diverse selectivity profiles.

### 1.1. Previous SAR Work and Evidence of PACAP_1__–__23_ Neuroprotection In Vitro

Several structure–activity relationship (SAR) studies have been carried out on PACAP to identify important pharmacophoric elements and sites prone to proteolytic cleavage [28,29,30,31]. C-terminal truncation studies revealed that the minimum sequence required to maintain adequate receptor binding is PACAP_1–23_, whereas studies investigating N-terminal deletions demonstrated that the 1st six residues are required to maintain agonist activity [30]. Due to the weak receptor binding profile of PACAP_1–23_, it was rarely investigated further for its neuroprotective potential [32]. However, in 2019, Chatenet and coworkers explored the in vitro functional activity and neuroprotective potential of PACAP_1__–__23_ in neuroblastoma cells [33]. They demonstrated that PACAP_1__–__23_ is capable of attenuating MPP^+^-induced apoptosis, mitochondrial dysfunction, and glutamate-induced excitotoxicity despite drastically reduced binding affinity at PAC1 [33]. In addition, they observed comparable potency between PACAP_1__–__23_ and PACAP_1__–__38_ in activating specific downstream signaling pathways. These data demonstrate that PACAP_1__–__23_ represents a potential candidate for the treatment of neurodegenerative diseases.

### 1.2. PACAP_1-23_ Glycopeptide Design Considerations

**Figure 1:**
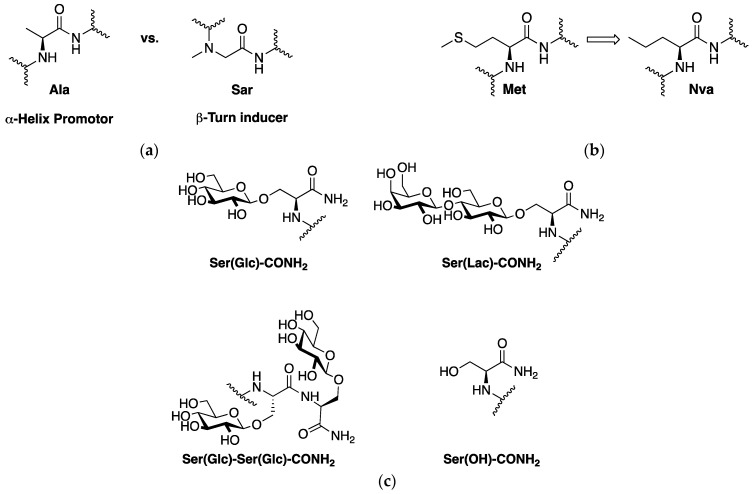
Strategic amino acid substitutions to fine-tune receptor selectivity and optimize stability and functional activity: (**a**) the bioactive conformation of our analogues can be fine-tuned by introducing amino acids into the 4th position that are biased towards either α-helical or β-turn-like conformations; (**b**) Met^17^ was replaced with norvaline (Nva) to enhance the stability of our glycopeptides. Methionine is easily oxidized and likely contributes to the low in vivo stability of native PACAP isoforms. (**c**) Various carbohydrate motifs were investigated in these studies to examine the effects on in vitro efficacy and potency of our analogues. Specifically, we examined serine, glucose, lactose, and two sequential serine glucoside residues as a lactose mimic.

Despite the neuroprotective potential of PACAP_1__–__23_, the native peptide itself is a poor drug candidate due to its rapid in vivo metabolism and limited blood–brain barrier (BBB) permeability. Furthermore, activation of the VPAC2 receptor may be undesirable in some contexts, making it important to pursue the discovery of selective agonists of the PAC1 receptor [34]. PACAP_1__–__38_ enters the brain through a selective transporter at the BBB endothelium known as peptide transporter system 6 (PTS-6), whereas PACAP_1–27_ appears to enter the brain via passive diffusion [35,36]. However, it is unknown if PACAP_1–23_ can penetrate the BBB like the native PACAP isoforms. Many strategies have been implemented to improve the pharmacokinetics and membrane permeability of peptides, including N-methylation, cyclization, lipidation, and PEGylation [37,38,39]. One strategy that is highly effective yet often overlooked is glycosylation. Several laboratories have demonstrated that glycosylation of peptides dramatically increases stability in vivo and enhances permeability of the BBB, which is evident through observing central effects and microdialysis in the striatum followed by mass spectrometry analysis following peripheral administration (*i.v.*, *i.p.*) [40,41,42,43,44,45]. Our laboratory has extensively investigated glycosylation as a means to address the concerns of BBB permeability and stability for many endogenous peptides including opioid peptides, angiotensin_1__–__7_, and PACAP [46,47,48,49,50]. In fact, we have shown that glycosylated PACAP analogues not only successfully penetrate the BBB, but that they also elicit potent neuroprotection in animal models of TBI, stroke, and PD [51,52]. Carbohydrates provide the necessary steric bulk to protect peptides from proteolytic degradation and modulate the amphipathicity of peptides such that it influences how they interact with biological membranes [40,43]. We hypothesized that converting relatively lipophilic peptides to amphipathic glycopeptides allows them to “hop” along the surface of cell membranes and, therefore, increases the probability that the glycopeptide will find and interact with the target receptor of interest. In the context of BBB permeability, we have hypothesized that glycopeptides can pass through the BBB via adsorptive transcytosis similarly to peptides with a high degree of positive charge [53]. However, the exact mechanism by which glycopeptides penetrate the BBB is still unknown. Considering our past successes in enhancing the in vivo stability and BBB penetrance of endogenous peptide hormones by glycosylation, particularly PACAP_1–27_ (unpublished results), we envisioned that applying this strategy to PACAP_1–23_ would provide analogues with enhanced stability and BBB penetrance. We incorporated various carbohydrate motifs (glucose, di-glucose, lactose, Figure 1) to determine which carbohydrate will provide the optimal balance between stability and in vitro functional activity in ongoing studies. The carbohydrates investigated in this study were chosen based on previously successful studies on glycosylated endogenous peptide scaffolds including angiotensin_1–7_, the enkephalins, and PACAP_1–27_ [40,43,48,51,52]. In addition to glycosylation of PACAP_1–23_, we set out to examine structural modifications predicted to increase selectivity for PAC1 and provide additional stability. We opted to replace Met^17^, which is susceptible to oxidation, with norvaline (Nva), a non-natural amino acid with an all-carbon side chain that cannot undergo oxidation. To fine-tune receptor selectivity, we chose to modify PACAP’s N-terminal region. The N-terminal region of PACAP exhibits high similarity to VIP (Table 1), but those similarities diverge at position 4. In PACAP, position 4 is occupied by Gly, which is a known β-turn inducer. In VIP, position 4 is occupied by Ala, which is well known as an α-helix stabilizer. Thus, it has been hypothesized that incorporation of amino acids capable of biasing the conformation of the N-terminus towards β-turns and α-helices would lead to selectivity for PAC1 or VPAC1/VPAC2, respectively. To this end, we prepared analogues containing either Ala, a known α-helix promoter, and *N*-methylglycine (Sar) (Figure 1), which has been shown to induce β-turn-like conformations. Herein, we present the synthesis and in vitro characterization of glycosylated analogues of PACAP_1–23_.

## 2. Results

### 2.1. Synthesis

**Figure 2:**
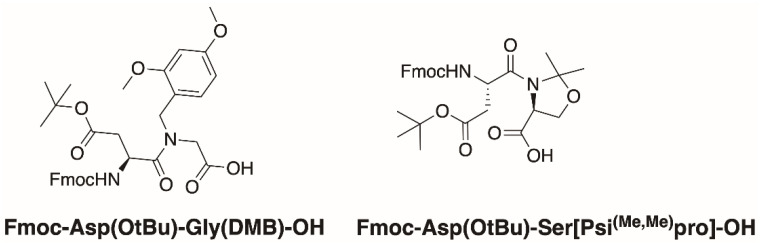
Amino acid building blocks utilized to suppress aspartimide formation.

The PACAP_1–23_ glycopeptide analogues were synthesized on a Rink amide MBHA resin (0.25 mmol scale, d.s. ~0.5 mmol/g) using the Prelude^®^ automated peptide synthesizer from Gyros Protein Technologies. The carbohydrate motifs were introduced as pre-assembled Fmoc protected serine glycoside buildings blocks, which were prepared via established methods in our laboratory utilizing minimally competent InBr_3_ catalysis [54]. Several different coupling protocols were utilized in this synthesis due to the fact that PACAP is considered a “difficult” peptide sequence [55]. PACAP’s relatively long length necessitates the use of stronger coupling reagents later on in the synthesis, and there are two motifs present that are highly prone to aspartimide formation (Asp^3^-Gly^4^ and Asp^8^-Ser^9^). Aspartimide formation involves the base-promoted cyclization of an aspartic acid side chain with the α-amino nitrogen of the preceding residue, with Gly, Ser, Thr, and Cys being the most problematic [56,57,58]. To circumvent this issue, we used dipeptide building blocks that suppress aspartimide formation. In the case of the Asp^8^-Ser^9^ motif, we utilized a pseudoproline dipeptide building block (Fmoc-DS, Figure 2), and for the Asp^3^-Gly^4^, we utilized a dimethoxybenzyl (DMB)-containing dipeptide (Fmoc-DG, Figure 2) [57]. The Fmoc-DS and Fmoc-DG dipeptide building blocks are unable to undergo aspartimide formation due to the alkyl protection of the amide nitrogens of the Ser and Gly motifs. In the case of Fmoc-DS, the serine amide nitrogen is locked into a pseudoproline ring that is cleaved upon treatment with TFA during resin cleavage. In the case of Fmoc-DG, the amide nitrogen of the Gly moiety is protected with a 2,4-dimethoxybenzyl group, which can be removed under the acidic of resin cleavage and global side chain deprotection. In addition to suppressing aspartimide formation, these specially protected dipeptide building blocks are also beneficial in that they minimize peptide–peptide aggregation on the resin, ultimately enhancing the efficiency of subsequent amino acid couplings.

**Figure 3:**
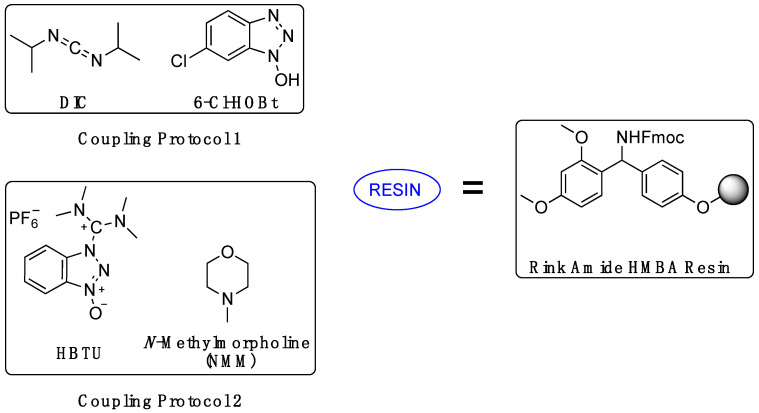
Resin and coupling protocols utilized in the synthesis.

**Scheme 1:**
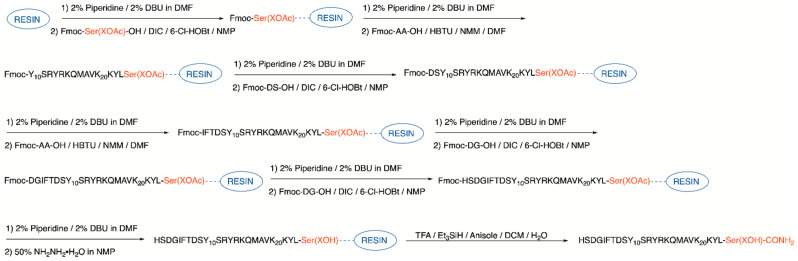
General synthetic scheme for glycopeptide preparation. The glycopeptides were constructed on a Rink amide HMBA resin. Coupling protocols using DIC/6-Cl-HOBt or HBTU/NMM were utilized depending on the identity of the amino acids. The acetate protecting groups in the glycoside moieties were cleanly removed using 50% H_2_N–NH_2_•H_2_O in NMP. Global side chain deprotection and resin cleavage were carried out using a cocktail consisting of TFA/Et_3_SiH/anisole/DCM/H_2_O.

With this plan in hand, we then set out to complete the glycopeptide assembly. First, the desired Fmoc protected glycosyl amino acid or Fmoc-Ser(OtBu)-OH was loaded onto the resin using equimolar amounts of Cl-HOBt and DIC in NMP (Scheme 1, Figure 3). The resin was then treated with a mixture of DIPEA/Ac_2_O in DCM (10%/10% *v*/*v*) to cap any unreacted sites on the resin. The next 14 residues (Tyr^10^-Leu^23^) were coupled utilizing a standard HBTU/N-methylmorpholine coupling protocol (Scheme 1). The Fmoc-DS dipeptide was coupled using the more reactive Cl-HOBt/DIC to introduce Asp^8^ and Ser^9^ due to increased steric hinderance. The relatively lipophilic tripeptide motif Ile^5^-Phe^6^-Thr^7^ was introduced using a modified HBTU/N-methylmorpholine coupling protocol where greater excesses of the coupling reagent and base were used to achieve complete coupling. The Fmoc-DG dipeptide was utilized in cases where the Asp^3^-Gly^4^ motif was present. In the cases of **5** and **6**, the Cl-HOBt/DIC coupling protocol was utilized to couple Ala^4^ or Sar^4^. As in the case of Fmoc-DS, the Cl-HOBt/DIC protocol was employed due to additional steric hinderance. The remaining amino acids were coupled using the Cl-HOBt/DIC in NMP protocol. After cleavage of the final Fmoc group on His^1^, the resin was treated with 50% NH_2_NH_2_•H_2_O in NMP to remove the carbohydrate acetate protecting groups. The crude peptides were precipitated in cold diethyl ether and purified by preparative HPLC. To our delight, the final purity of the shorter PACAP_1–23_ glycopeptides (≥98%) was superior to PACAP_1–27_ glycopeptides previously prepared in our laboratory [52]. The retention time data of the pure glycopeptides are reported in Table 2. The molecular masses of the glycopeptide analogues were determined by ESI-MS, resulting in agreement with the theoretical masses calculated from the predicted primary structures. The most predominant peaks in the mass spectra were in the +4 or +5 charge state, which was expected due to the presence of a significant number of basic residues in PACAP’s primary sequence (Table 2) (Supplementary Materials).

### 2.2. In Vitro Characterization

Following synthesis, purification, and characterization, the glycopeptides were assessed for their ability to stimulate cAMP in CHO cells individually expressing the PAC1, VPAC1, and VPAC2 receptors (Table 3). Introduction of a serine with no carbohydrate (**1**) yielded a compound with low nanomolar activity at PAC1 and VPAC1, but activity at VPAC2 was greatly diminished compared to the control. The incorporation of a serine glucoside moiety at the C-terminus (**2**) did not alter activity at VPAC2 significantly, but potency at PAC1 was greatly enhanced. When a second serine glucoside was introduced to give the di-glucoside-containing compound **3**, activity at VPAC2 dropped sharply, while potency and efficacy were maintained at PAC1 and VPAC1, albeit with reduced potency at PAC1 compared to **2**. The compounds containing serine lactoside motifs at the C-terminus (**4**, **5**, **6**) were PAC1/VPAC1 selective, with slightly greater potency at PAC1 over VPAC1. Additionally, these compounds exhibited extremely weak activity at the VPAC2 receptor, similarly to the serine–glucoside and serine–di-glucoside compounds.

## 3. Discussion

Our group has previously investigated the glycosylation of native PACAP_1–27_ as a strategy to improve its PK properties while maintaining its intrinsic potency and efficacy in a rodent model of PD [51,52]. Even shorter versions of this hormone (e.g., PACAP_1–23_) demonstrate neuroprotective activity in vitro [33], showing potency similar to native PACAP_1–38_ in the attenuation of glutamate-induced cytotoxicity and MPP^+^-induced cell death, suggesting that shorter PACAP analogues could be viable alternatives to the longer hormones. This is advantageous for several reasons. First, PACAP_1–27_ and PACAP_1–38_ are quite long sequences and difficult to synthesize, and the shorter PACAP glycosides are marginally easier to prepare in their pure state. Six glycopeptide analogues were prepared in satisfactory yields and higher purity than our previously prepared PACAP glycopeptide analogues (Supplementary Materials, [52]). The modifications we introduced include various glycoside motifs at the C-terminus (glucose, di-glucose, lactose) to enhance stability and BBB transport and N-terminal substitutions in position 4 (Ala, *N*-methyl glycine) to fine-tune receptor selectivity. To our delight, the introduction of carbohydrate-bearing amino acids into the C-termini of our compounds did not negatively impact functional activity. PAC1/VPAC1 selectivity was maintained, and activity at VPAC2 was drastically reduced. Interestingly, functional activity at VPAC2 was drastically reduced upon introduction of a di-glucoside motif (**3**), or a lactose residue (**4**, **6**). However, **5** seems to be an exception to this trend, but this was expected considering the Ala^4^ substitution, which is known to enhance selectivity for the VPAC1/VPAC2 receptors. This decrease in activity at VPAC2 may be attributed to disfavored binding interactions between the carbohydrate moiety and the extracellular domain (ECD) of the receptor, but this has not been confirmed. Thus, further exploration into the origin of carbohydrate-mediated decreases in VPAC2 selectivity are warranted. Although the Sar^4^ substitution yielded a compound that was PAC1 selective, potency was decreased by a considerable amount compared to the other compounds. The best compound in the series was determined to be the mono-glucoside **2**, with ~58-fold selectivity for PAC1 over VPAC1 and ~886-fold selectivity for PAC1 over VPAC2. However, **3** (serine di-glucoside) and **4** (serine lactoside) also exhibited ideal functional activity and receptor selectivity profiles and are scaffolds worth pursuing in future PACAP glycopeptide drug discovery efforts. Ongoing studies will elucidate the effects of the substitutions investigated in this study on the in vitro and in vivo stability of our PACAP_1__–23_ glycopeptide analogues.

## 4. Materials and Methods

### 4.1. Glycopeptide Synthesis and Purification

Fmoc-based solid phase peptide synthesis (SPPS) was used to synthesize the 6 drug candidates on Rink resin to produce the C-terminal amides. Acetate removal from the glycosides was accomplished “on resin” with hydrazine hydrate (H_2_N–NH_2_•H_2_O) per previously published methods [47]. Three distinct coupling methods were used to assemble the glycopeptide backbone that contains two “difficult sequences” that can form aspartimides, leading to α to β amide migration. This complication was avoided by the use of dipeptides with pre-formed amide linkages to each aspartic acid residue [57,59,60].

#### 4.1.1. General

Peptide synthesis was performed on a Prelude^®^ automated peptide synthesizer (Gyros Protein Technologies, Tucson, AZ, USA). Synthesis was performed either in an automated fashion or semi-manually where reagents were loaded into the reaction vessels using a syringe. The resin was agitated (mixed) using a steady flow of argon. The washing steps with DMF and DCM were performed for 2 min each.

#### 4.1.2. Rink Amide Resin Preparation

A sample of 0.25 mmol of Rink amide-MBHA resin (0.6 g) resin was placed in a 45 mL reaction vessel and swelled in DMF for 1 h. Fmoc removal was achieved by addition of a solution containing 2%DBU-3%piperidine in DMF (6 mL) and mixing for 4 min. The mixture was then drained, and the resin was washed once with 6mL of DMF. Fmoc removal was then repeated for an additional 8 min followed by 6 DMF washes (6 mL, 2 min).

#### 4.1.3. Serine or Glycosyl Amino Acid Loading

A sample of 0.20 mmol (0.8 eq.) of the desired first amino acid Fmoc-Ser(tBu)-OH, Fmoc-Ser(Glc(OAc)_4_)-OH, or Fmoc-Ser(Lac(OAc)_7_)-OH) and 0.2 mmol (0.8 eq.) 6-Cl-HOBt were placed into a vial and dissolved in 4 mL NMP. A sample of 0.2 mmol (0.8 eq.) of DIC was then added into the solution. The mixture was vortexed and/or sonicated for 1 min and then added to the resin. The reaction mixture was mixed overnight for 16 h. The mixture was diluted with DMF (10 mL) and drained immediately. Then, the resin was washed 6 times with DMF (6 mL) and then 6 times with DCM (6 mL). The unreacted NH_2_ sites on the resin were then capped with a solution of 10% N,N-diisopropylethylamine and 10% Ac_2_O in 8mL DCM. This reaction was allowed to proceed for 1 h. The resin was then washed 6 times with DCM (6 mL) and then washed 4 times with DMF (6 mL) to prepare the resin for the next automated steps.

#### 4.1.4. Loading of Additional Fmoc-Ser(Glc(OAc)_4_)-OH (**3** Only)

Samples of 0.5 mmol (2.0 eq.) of Fmoc-Ser(Glc(OAc)_4_)-OH and 0.5 mmol (2.0 eq.) 6-Cl-HOBt were placed into a vial and dissolved in 4 mL NMP. A sample of 0.5 mmol (2.0 eq.) of DIC was then added into the solution. The mixture was vortexed and/or sonicated for 1 min and then added to the resin. The reaction mixture was mixed overnight for 16 h. The mixture was diluted with DMF (10 mL) and drained immediately. Then, the resin was washed 6 times with DMF (6 mL).

#### 4.1.5. Prelude^®^ Automated Synthesis

The Leu^23^-Tyr^10^ amino acid series was prepared using the automated SPPS feature on the Prelude^®^ automated peptide synthesizer. The Fmoc group was removed as described above and a solution containing the desired Fmoc amino acid (2 equivalents), HBTU (2 equivalents) and *N*-methylmorpholine (10 equivalents) was loaded to the resin. The reaction mixture was mixed for 30 min followed by a single DMF wash (6 mL). The coupling reaction was repeated a second time for 30 min, and the resin was then washed 6 times with DMF (6 mL). Subsequent deprotection and coupling cycles were then performed up to tyrosine^10^.

#### 4.1.6. Manual Loading of DS Dipeptide

The Fmoc group was initially removed as described above. Then, 0.3 mmol of Fmoc-DS-OH or Fmoc-DG-OH (1.2 equiv.) and 0.3 mmol of 6-Cl-HOBt (1.2 equiv.) were added to a vial and dissolved in 4 mL of NMP. A sample of 0.3 mmol of DIC (1.2 equiv.) was then added to the solution. The mixture was vortexed and/or sonicated for 1 min and then added to the resin. The reaction mixture was mixed for 16 h. The mixture was diluted with DMF (10 mL) and drained immediately. Then, the resin was washed 6 times with DMF (6 mL).

#### 4.1.7. Automated Addition of IFT

The Ile^5^-Thr^7^ amino acid series was prepared using the automated SPPS feature on the Prelude^®^ automated peptide synthesizer. The Fmoc group was removed as described above and a solution containing the desired Fmoc amino acid (4 equivalents), HBTU (4 equivalents), and *N*-methylmorpholine (16 equivalents) in 10 mL of DMF was loaded into the resin. The reaction mixture was mixed for 30 min followed by a single DMF wash (10 mL). The coupling reaction was repeated a second time for 30 min, and the resin was then washed 6 times with DMF. Subsequent deprotection and coupling cycles were then performed up to isoleucine^5^.

#### 4.1.8. Manual Loading of DG Dipeptide

The Fmoc group was initially removed as described above. Then, 0.5 mmol Fmoc-DG-OH (2.0 equiv.) and 0.5 mmol of 6-Cl-HOBt (2.0 equiv.) were added to a vial and dissolved in 4 mL of NMP. A sample of 0.5 mmol of DIC (2.0 equiv.) was then added to the solution. The mixture was vortexed and/or sonicated for 1 min and then added to the resin. The reaction mixture was then mixed for 6 h. The mixture was diluted with DMF (10 mL) and drained immediately. Then, the resin was washed 6 times with DMF (6 mL).

#### 4.1.9. Manual Loading of Fmoc-Ala-OH (**5**) or Fmoc-Sar-OH (**6**)

The Fmoc group was initially removed as described above. Then, 1.0 mmol of amino acid (4 equiv.) and 1.0 mmol of 6-Cl-HOBt (4 equiv.) were added to a vial and dissolved in 4 mL of NMP. A sample of 1.0 mmol of DIC (4 equiv.) was then added to the solution. The mixture was vortexed for 1 min and then added to the resin. The reaction mixture was mixed for 2 h. The mixture was diluted with DMF (10 mL) and drained immediately. Then, the resin was washed 6 times with DMF (6 mL).

#### 4.1.10. Manual Loading of Remaining Amino Acids Fmoc-Asp(tBu)-OH (**5** and **6** Only), Fmoc-Ser(tBu)-OH, and Fmoc-His(Trt)-OH

The Fmoc group was initially removed as described above. Then, 1.0 mmol amino acid (4 equiv.) and 1.0 mmol of 6-Cl-HOBt (4 equiv.) were added to a vial and dissolved in 4 mL of NMP. A sample of 1.0 mmol of DIC (4 equiv.) was then added to the solution. The mixture was vortexed for 1 min and then added to the resin. The reaction mixture was mixed for 60 min. The mixture was diluted with DMF (10 mL) and drained immediately. Then, the resin was washed 6 times with DMF (6 mL). After addition of the final amino acid, the Fmoc group was removed as described above.

#### 4.1.11. Acetyl Cleavage

One hundred and twenty milliliters of a 50% solution containing NH_2_NH_2_ × H_2_O in NMP (10 mL per reaction vessel) was prepared and added to the resin. The solution was mixed overnight for 16 h. The solution was then drained, and a second 10 mL portion of 50% NH_2_NH_2_ x H_2_O was added to each reaction vessel. This solution was mixed for an additional 2 h. The 50% NH_2_NH_2_ x H_2_O was then drained, and the resin was washed 8 times with DMF (10 mL), 8 times with DCM (10 mL), and dried under vacuum for 3 h.

#### 4.1.12. Cleavage from the Resin and Global Side Chain Deprotection

The dried resin was treated with an acidic cleavage cocktail containing TFA, DCM, H_2_O, triethylsilane, and anisole (90:10:2:3:0.5). The resin was mixed for 1 h, and the solution was collected in a 45 mL centrifuge tube. The cleavage step was repeated 2 more times for 10 min periods. The combined fractions were slowly evaporated over a stream of argon until the peptide began to crash out. Cold ether (~40 mL) was then added to precipitate the peptide and the mixture was centrifuged for 10 min at 5 G. The ether layer was decanted off and ether (~40 mL) was added to the crude peptide and centrifuged once more. This process was repeated for a third time. After decanting the ether layer, the crude peptide was dried under vacuum overnight.

#### 4.1.13. HPLC Purification and Characterization of Peptides

These crude samples were then purified on a Gilson system with a UV detector (at 280 nm) using a Vydak C18 preparative reversed-phase column (250 mm × 50 mm) using a gradient of 5–80% CH_3_CN vs. 0.1% CF_3_COOH in H_2_O over 60 min to give the glycopeptides in pure form, which were assessed for purity by analytical HPLC (Inspire C18 5 μm 250 mm × 4.6 mm column) on a Varian LC with a diode array detector system (at 280 nm) employing the same gradient over a period of 25 min.

The pure fractions obtained from preparative HPLC purification were frozen at −80 °C and then lyophilized to afford the pure peptides as white and fluffy solids. The pure peptides were then characterized using mass spectrometry (ESI-MS) (University of Arizona Analytical and Biological Mass Spectrometry Core Facility, Tucson, AZ, USA).

### 4.2. Cell Culture

CHO cells stably expressing cloned PAC1, VPAC1, and VPAC2 were produced by electroporation with human PAC1/VPAC1/VPAC2 N-3xHA tag cDNA constructs (GeneCopoeia). Cells were grown on 10 cm dishes in DMEM/F-12 50/50 mix w/L-glutamine and 15 mM HEPES (Corning) containing 10% heat inactivated fetal bovine serum, 100 units/mL penicillin, 100 μg/mL streptomycin, and 500 μg/mL G418 under 5% CO_2_ at 37 °C. The cells were enriched into high expressing populations using flow cytometry, selecting the top ~2% of expressing cells.

### 4.3. cAMP Accumulation Assay

At ~80% confluence, cells were plated into 96-well plates (20,000 cells/well) and grown in the same medium and conditions as described above for 24 h. The cells were then serum starved for 4 h. After a 20 min incubation at 37 °C with 500 μM 3-Isobutyl-1-methylxanthine (IBMX), serum-free medium containing 500 μM IBMX and the appropriate agonists was added and then incubated for 10 min at 37 °C. The reaction was terminated by removing the medium and adding 60 μL of ice-cold assay buffer (50 mM Tris-HCl pH 7.4, 100 mM NaCl, 5 mM ethylenediaminetetraacetic acid (EDTA)). Plates were sealed with boiling mats and then boiled at 95 °C for 10 min. Plates were then centrifuged at 4000 rpm, 4 °C, for 10 min to remove debris. Fifty microliters of lysate was transferred to a 96-well plate. Lysate was incubated with ~1 pmol ^3^H-cAMP (PerkinElmer), and 7 μg protein kinase A (Sigma-Aldrich, St. Louis, MI, USA) with 0.05% bovine serum albumin (BSA). The assay was incubated at room temperature for 1 h. The reactions were then harvested onto GF/B filter plates (PerkinElmer) via rapid filtration by a 96-well plate Cell Harvester (Brandel) and washed 3 times with ice-cold water. Filter plates were dried, 40μL of Microscint-PS scintillation cocktail was added to each well, and then counted in a TopCount or Microbeta2 (PerkinElmer) microplate scintillation counter.

## 5. Conclusions

The shorter glycosidic PACAP compounds display unique receptor profiles with greatly reduced activity for the VPAC2 receptor while retaining PAC1 and VPAC1 agonist activity. These compounds may be useful in the treatment of traumatic brain injury, stroke, Parkinsonism, or other progressive disorders.

## 6. Patents

The novel PACAP glycosides have been patented by Tech Launch Arizona, the University of Arizona, University Services Annex West, 4th Floor, 220 West 6th Street, Tucson, AZ 85721.

## Figures and Tables

**Table 1:** Structures of relevant peptides/glycopeptides for this study.

Compound	Structure ^1^
PACAP_1–38_	HSDGIFTDSY_10_SRYRKQMAVK_20_KYLAAVLGKRYKQRVKNK-CONH_2_
PACAP_1–27_	HSDGIFTDSY_10_SRYRKQMAVK_20_KYLAAVL-CONH_2_
PACAP_1–23_	HSDGIFTDSY_10_SRYRKQMAVK_20_KYL-CONH_2_
VIP	HSDAVFTDNY_10_TRLRKQMAVK_20_KYLNSILN-CONH_2_
**1**	HSDGIFTDSY_10_SRYRKQÑAVK_20_KYL-Ser(OH)-CONH_2_
**2**	HSDGIFTDSY_10_SRYRKQÑAVK_20_KYL-Ser(Glc)-CONH_2_
**3**	HSDGIFTDSY_10_SRYRKQÑAVK_20_KYL-Ser(Glc)-Ser(Glc)-CONH_2_
**4**	HSDGIFTDSY_10_SRYRKQÑAVK_20_KYL-Ser(Lac)-CONH_2_
**5**	HSDAIFTDSY_10_SRYRKQÑAVK_20_KYL-Ser(Lac)-CONH_2_
**6**	HSD∑IFTDSY_10_SRYRKQÑAVK_20_KYL-Ser(Lac)-CONH_2_

^1^ Ñ = Νorvaline, Σ = Sarcosine.

**Table 2:** Characterization of synthetic PACAP_1–23_ glycopeptides.

Compound	Molecular Formula	Calculated Mass	^1^ Experimental Mass →		^2^ HPLC Ret. Time (min)
** 1 **	C_128_H_199_N_37_O_37_	2846.48	570.71 (M+5H)^5+^	2848.55	13.98
** 2 **	C_134_H_209_N_37_O_42_	3008.54	603.09 (M+5H)^5+^	3010.45	13.60
** 3 **	C_143_H_224_N_38_O_49_	3257.62	652.76 (M+5H)^5+^	3258.80	13.24
** 4 **	C_140_H_219_N_37_O_47_	3170.59	793.93 (M+4H)^4+^	3171.72	13.59
** 5 **	C_141_H_221_N_37_O_47_	3184.60	638.09 (M+5H)^5+^	3185.45	13.48
** 6 **	C_141_H_221_N_37_O_47_	3184.60	638.01 (M+5H)^5+^	3185.45	13.42

^1^ *m*/*z* values evaluated by ESI-MS. ^2^ HPLC conditions: 5–80% CH_3_CN vs. 0.1% CF_3_COOH in H_2_O over 60 min.

**Table 3:** In vitro cAMP stimulation on PAC1-CHO, VPAC1-CHO, and VPAC2-CHO cells in the presence of PACAP_1–23_. Glycopeptide analogues.

Drug Candidate	PAC1		VPAC1		VPAC2	
	EC_50_ (nM)	E_MAX_ (%)	EC_50_ (nM)	E_MAX_ (%)	EC_50_ (nM)	E_MAX_ (%)
**PACAP_1–27_**	20, 9.0	100, 100	30, 6.5	100, 100	71, 29	100, 100
**1**	2.2	198	5.2	42	571	111
**2**	0.64	181	37	104	567	110
**3**	1.9	152	26	147	7369	128
**4**	1.8	174	21	118	1943	169
**5**	7.0	181	38	124	321	139
**6**	67	134	195	107	NC	NC

NC = No convergence.

## Data Availability

Data of the compounds are available from the authors.

## Supplementary Materials

The following are available online. Table S1: Glycopeptide yields. The original analytical HPLC chromatograms and ESI-MS spectra are available in the supplementary material. The binding curves corresponding to the in vitro cAMP accumulation data are also available in the supplementary material.

## Sample Availability

Not available.
