# Supplementary material for: Synthesis and In Vitro Characterization of Glycopeptide Drug Candidates Related to PACAP1–23
Source: Molecules. 2021 Aug 14;26(16):4932. doi: 10.3390/molecules26164932 (PMC8401035; doi:10.3390/molecules26164932)
Supplement: Supplementary file 1 [file molecules-26-04932-s001.zip › molecules-1330908-SI.pdf]

Supplemental Information

Synthesis and *In Vitro* Characterization of Glycopeptide Drug Candidates Related to PACAP$_{1-23}$

Christopher R. Apostol[1], Parthasaradhireddy Tanguturi[3], Lajos Z. Szabò[1], Daniel Varela[2], Thiago Gilmartin[2], John M. Streicher[3], and Robin Polt[1*]

[1] Department of Chemistry and Biochemistry, BIO5, The University of Arizona, 1306 E. University Blvd, Tucson, AZ 85721; caposto1@email.arizona.edu
[2] Facultat de Quìmica Tarragona, Univeritat Rovera I Virgili, Barcelona, Spain
[3] Department of Pharmacology, College of Medicine, The University of Arizona, 1501 N. Campbell Ave, Tucson, AZ 85724
* polt@u.arizona.edu; +01 (520) 370-2654

Glycopeptide Yields

Compound	Yield on 0.25 mmol scale (mg)	%Yield
**CRA_DV_3022 (1)**	25.7	3.6
**CRA_DV_3023 (2)**	23.5	3.1
**CRA_DV_3024 (3)**	7.7	0.9
**CRA_TG_3025 (4)**	36	4.5
**CRA_TG_3026 (5)**	19.7	2.5
**CRA_TG_3027 (6)**	31.2	3.9

Analytical HPLC Data

CRA_DV 3022 (1)

Data File: c:\star\data\chris a\pacap 23mers\23mers
Channel: 2 = 280.00 nm RESULTS
Sample ID: CRA_DV_3022 pure
Operator (Inj): polt lab
Injection Date: 04/26/2021 04:16:13 PM
Injection Method: c:\star\method\lajos\chris30min.mth
Run Time (min): 37.013
Workstation:
Instrument (Inj): varian

Operator (Calc): polt lab
Calc Date: 04/26/2021 05:14:19
Times Calculated: 2
Calculation Method: c:\docume~1\poltlab\locals~1\temp\~2ls1
Instrument (Calc): varian
Run Mode: Analysis
Peak Measurement: Peak Area
Calculation Type: Percent
Calibration Level: N/A
Verification Tolerance: N/A

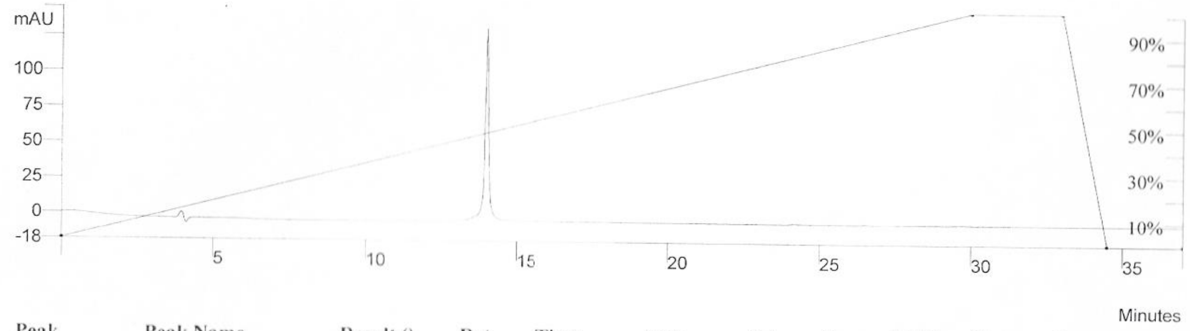

Peak No	Peak Name	Result ()	Ret. Time (min)	Time Offset (min)	Area (counts)	Rel Ret Time	Sep. Code	Width 1/2 (sec)	Status Codes	Group
1		100.0000	13.984	0.000	6515282	0.00	BB	7.0		0
	Totals	100.0000		0.000	6515282					

CRA_DV 3023 (2)

Data File: c:\star\data\chris a\pacap 23mers\23mers
Channel: 2 = 280.00 nm RESULTS
Sample ID: CRA_DV_3023 pure
Operator (Inj): polt lab
Injection Date: 04/26/2021 04:56:34 PM
Injection Method: c:\star\method\lajos\chris30min.mth
Run Time (min): 37.013
Workstation:
Instrument (Inj): varian

Operator (Calc): polt lab
Calc Date: 04/27/2021 09:59:52
Times Calculated: 2
Calculation Method: c:\docume~1\poltlab\locals~1\temp\~2ls1
Instrument (Calc): varian
Run Mode: Analysis
Peak Measurement: Peak Area
Calculation Type: Percent
Calibration Level: N/A
Verification Tolerance: N/A

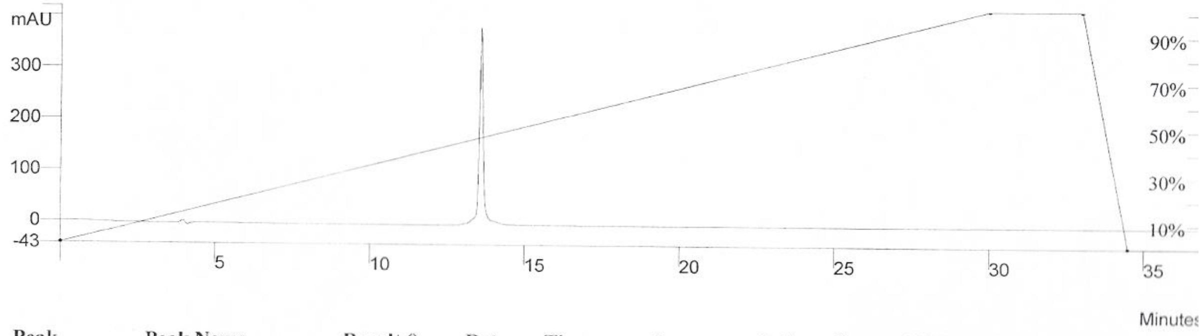

Peak No	Peak Name	Result ()	Ret. Time (min)	Time Offset (min)	Area (counts)	Rel Ret Time	Sep. Code	Width 1/2 (sec)	Status Codes	Group
1		100.0000	13.595	0.000	18338214	0.00	BB	7.2		0
	Totals	100.0000		0.000	18338214					

CRA_DV 3024 (3)

Data File:	c:\star\data\chris a\pacap 23mers\23mers	Operator (Calc):	polt lab
Channel:	2 = 280.00 nm RESULTS	Calc Date:	04/27/2021 10:00:37
Sample ID:	CRA_DV_3024 pure	Times Calculated:	2
Operator (Inj):	polt lab	Calculation Method:	c:\docume~1\poltlab\locals~1\temp\~2ls1
Injection Date:	04/26/2021 06:17:15 PM	Instrument (Calc):	varian
Injection Method:	c:\star\method\lajos\chris30min.mth	Run Mode:	Analysis
Run Time (min):	37.013	Peak Measurement:	Peak Area
Workstation:		Calculation Type:	Percent
Instrument (Inj):	varian	Calibration Level:	N/A
		Verification Tolerance:	N/A

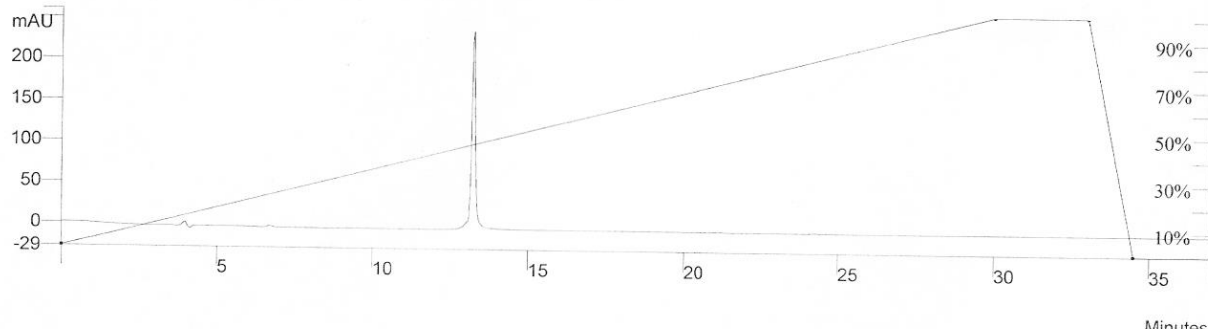

Peak No	Peak Name	Result ()	Ret. Time (min)	Time Offset (min)	Area (counts)	Rel Ret Time	Sep. Code	Width 1/2 (sec)	Status Codes	Group
1		100.0000	13.235	0.000	10758523	0.00	BB	7.0		0
	**Totals**	**100.0000**		**0.000**	**10758523**					

CRA_TG 3025 (4)

Data File:	c:\star\data\chris a\pacap 23mers\23mers	Operator (Calc):	polt lab
Channel:	2 = 280.00 nm RESULTS	Calc Date:	04/26/2021 02:05:20
Sample ID:	CRA_TG_3025 pure	Times Calculated:	2
Operator (Inj):	polt lab	Calculation Method:	c:\docume~1\poltlab\locals~1\temp\~cra_
Injection Date:	04/26/2021 12:54:31 PM	Instrument (Calc):	varian
Injection Method:	c:\star\method\lajos\chris30min.mth	Run Mode:	Analysis
Run Time (min):	37.013	Peak Measurement:	Peak Area
Workstation:		Calculation Type:	Percent
Instrument (Inj):	varian	Calibration Level:	N/A
		Verification Tolerance:	N/A

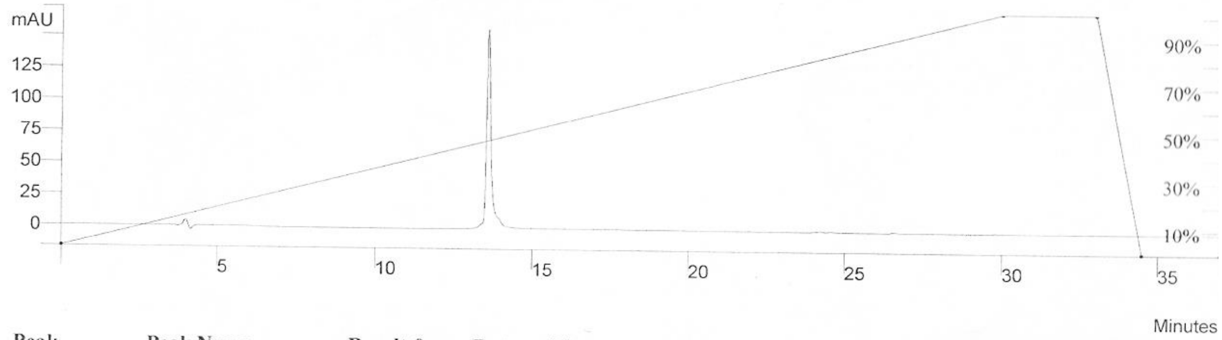

Peak No	Peak Name	Result ()	Ret. Time (min)	Time Offset (min)	Area (counts)	Rel Ret Time	Sep. Code	Width 1/2 (sec)	Status Codes	Group
1		100.0000	13.587	0.000	8293504	0.00	BB	7.8		0
	**Totals**	**100.0000**		**0.000**	**8293504**					

CRA_TG 3026 (5)

Data File:	c:\star\data\chris a\pacap 23mers\23mers	Operator (Calc):	polt lab
Channel:	2 = 280.00 nm RESULTS	Calc Date:	04/26/2021 02:22:18
Sample ID:	CRA_TG_3026 pure	Times Calculated:	2
Operator (Inj):	polt lab	Calculation Method:	c:\docume~1\poltlab\locals~1\temp\~cra_
Injection Date:	04/26/2021 01:34:52 PM	Instrument (Calc):	varian
Injection Method:	c:\star\method\lajos\chris30min.mth	Run Mode:	Analysis
Run Time (min):	37.013	Peak Measurement:	Peak Area
Workstation:		Calculation Type:	Percent
Instrument (Inj):	varian	Calibration Level:	N/A
		Verification Tolerance:	N/A

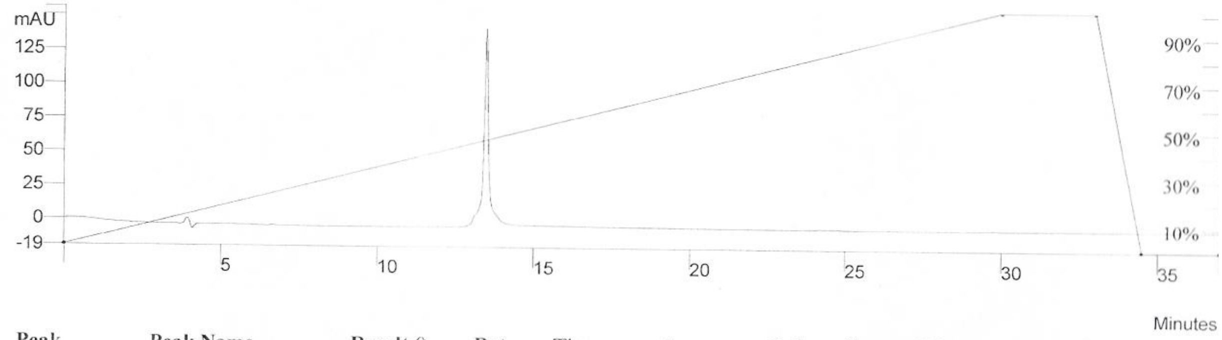

Peak No	Peak Name	Result ()	Ret. Time (min)	Time Offset (min)	Area (counts)	Rel Ret Time	Sep. Code	Width 1/2 (sec)	Status Codes	Group
1		100.0000	13.477	0.000	8895272	0.00	BB	8.0		0
	**Totals**	**100.0000**		**0.000**	**8895272**					

CRA_TG 3027 (6)

Data File:	c:\star\data\chris a\pacap 23mers\23mers	Operator (Calc):	polt lab
Channel:	2 = 280.00 nm RESULTS	Calc Date:	04/26/2021 02:58:34
Sample ID:	CRA_TG_3027 pure	Times Calculated:	2
Operator (Inj):	polt lab	Calculation Method:	c:\docume~1\poltlab\locals~1\temp\~cra_
Injection Date:	04/26/2021 02:15:13 PM	Instrument (Calc):	varian
Injection Method:	c:\star\method\lajos\chris30min.mth	Run Mode:	Analysis
Run Time (min):	37.013	Peak Measurement:	Peak Area
Workstation:		Calculation Type:	Percent
Instrument (Inj):	varian	Calibration Level:	N/A
		Verification Tolerance:	N/A

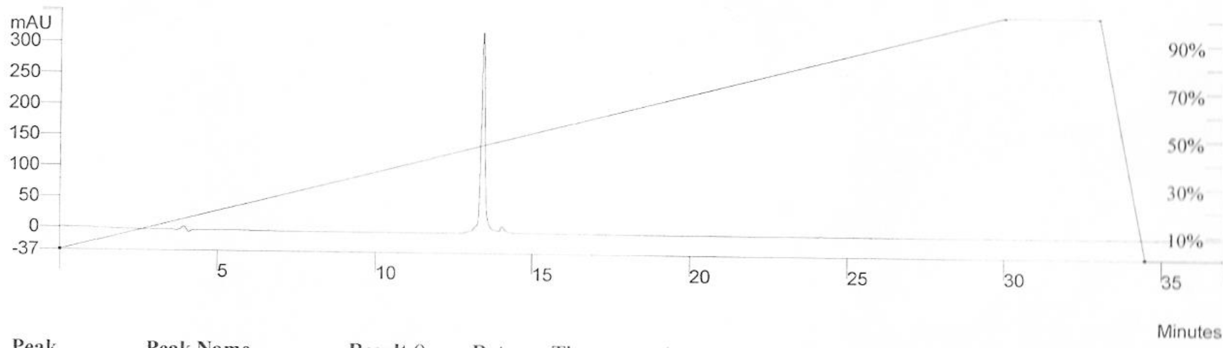

Peak No	Peak Name	Result ()	Ret. Time (min)	Time Offset (min)	Area (counts)	Rel Ret Time	Sep. Code	Width 1/2 (sec)	Status Codes	Group
1		97.8103	13.424	0.000	15267866	0.00	BB	7.2		0
2		2.1897	14.045	0.000	341804	0.00	TS	0.0		0
	Totals	100.0000		0.000	15609670					

Mass Spectra

CRA_DV 3022 (1)

Generic Display Report

Analysis Info

Analysis Name	D:\Data\2021_04\CRA-4110540\CRA_DV_3022_02.d	Acquisition Date	4/28/2021 3:47:01 PM
Method	Tuning Mix.m	Operator	KK
Sample Name	CRA_DV_3022	Instrument	amaZon SL
Comment	Dissolved in 200 ul 50%ACN, then 2x dil with 50%ACN and a few drops of 0.3%FA		

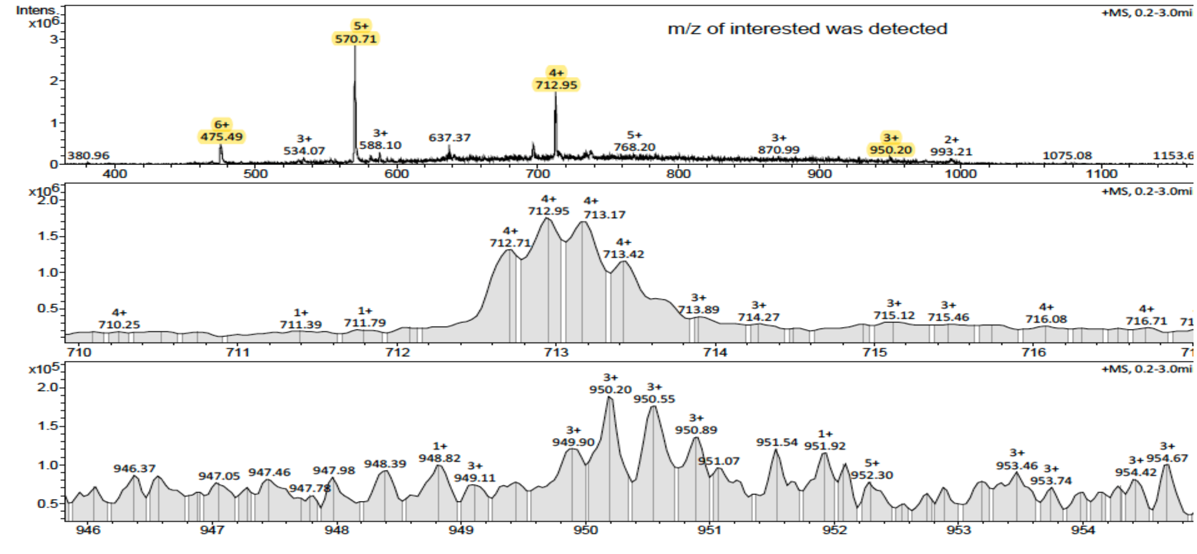

Bruker Compass DataAnalysis 4.2 printed: 4/28/2021 4:01:45 PM by: KK Page 1 of

CRA_DV 3023 (2)

Generic Display Report

**Analysis Info**		Acquisition Date	4/28/2021 4:20:13 PM
Analysis Name	D:\Data\2021_04\CRA-4110540\CRA_CRA_DV_3023.d		
Method	Tuning Mix.m	Operator	KK
Sample Name	CRA_DV_3023	Instrument	amaZon SL
Comment	Dissolved in 200 ul 50%ACN, then 2x dil with 50%ACN and a few drops of 0.3%FA. Ultrascan		

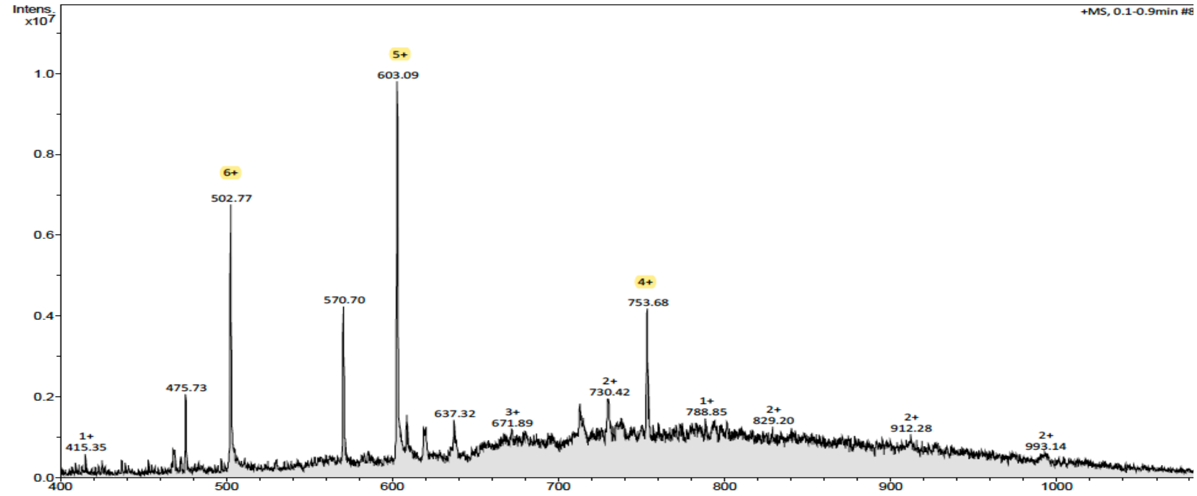

Bruker Compass DataAnalysis 4.2 printed: 4/30/2021 12:38:10 PM by: KK Page 1 of 1

CRA_DV 3024 (3)

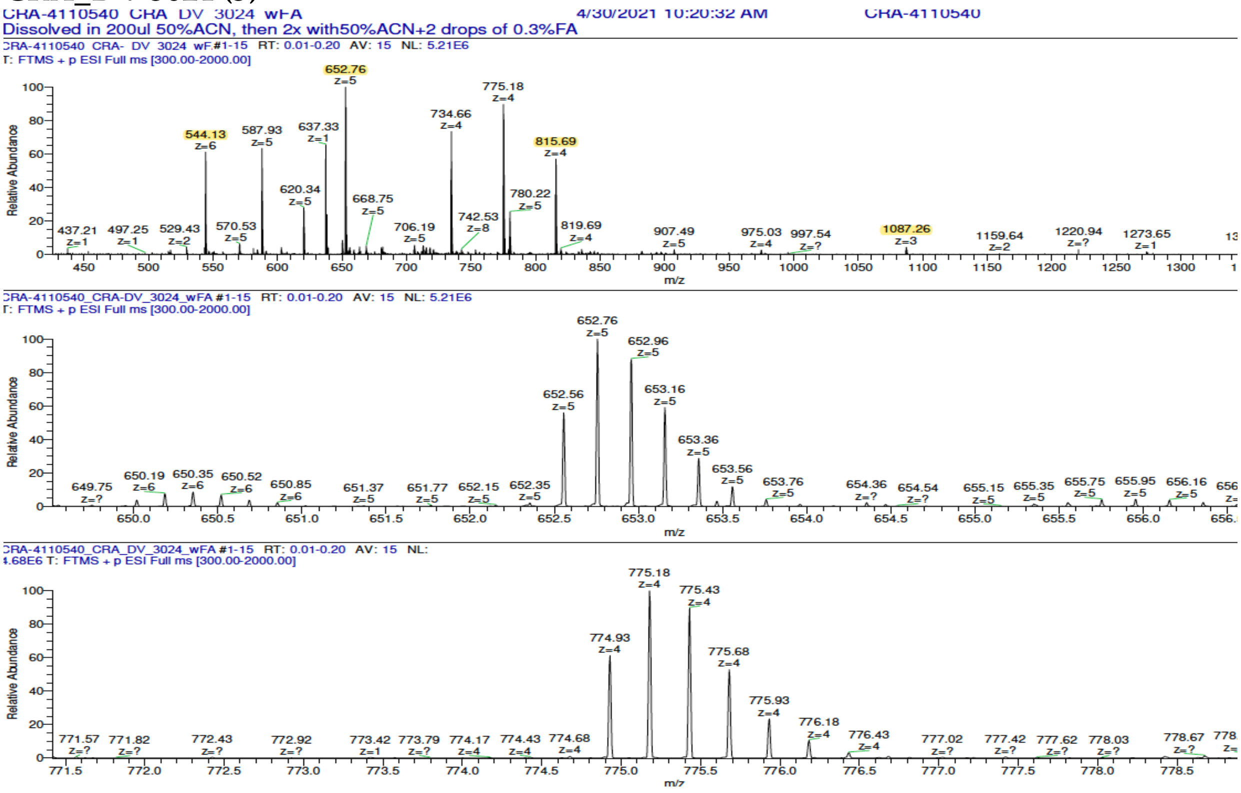

CRA_TG 3025 (4)

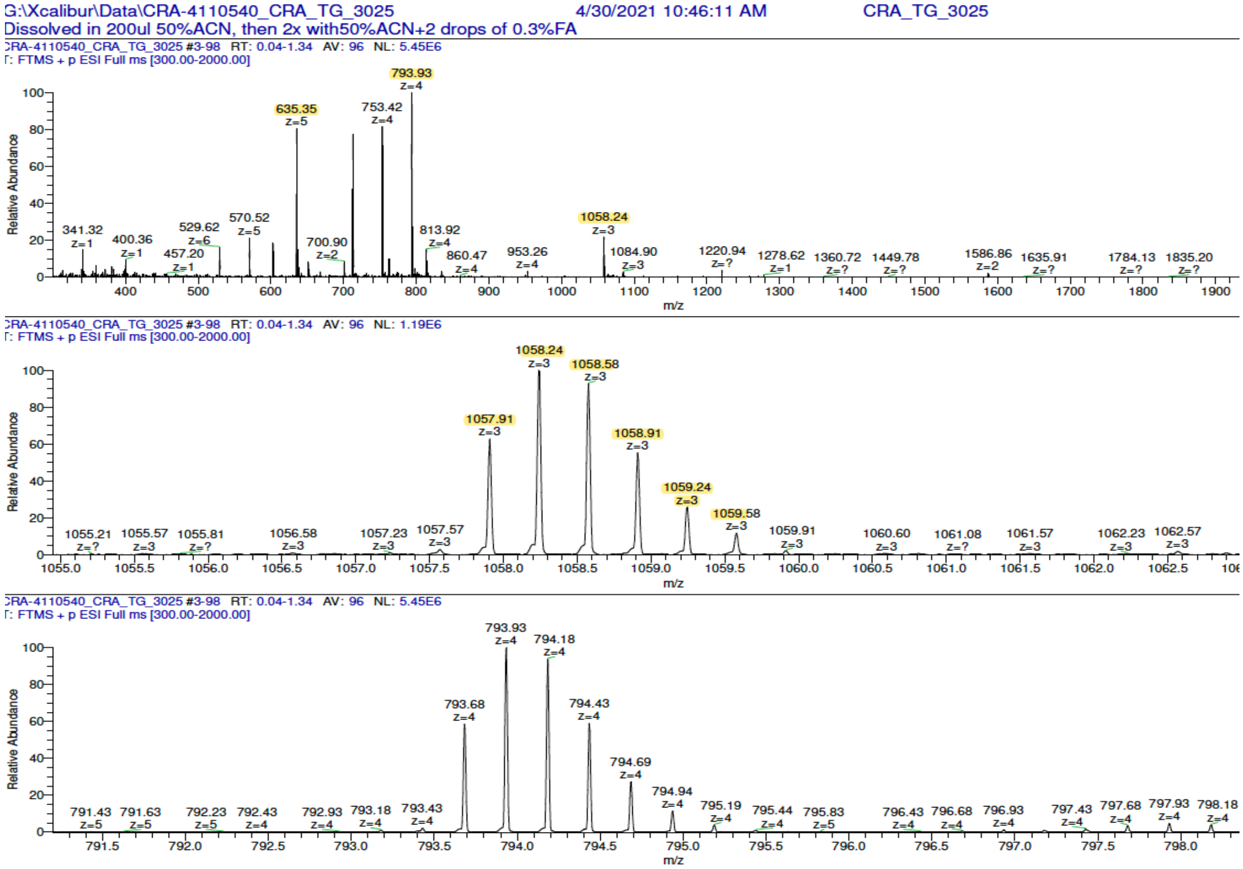
G:\Xcalibur\Data\CRA-4110540_CRA_TG_3025
4/30/2021 10:46:11 AM
CRA_TG_3025
Dissolved in 200ul 50%ACN, then 2x with50%ACN+2 drops of 0.3%FA
CRA-4110540_CRA_TG_3025 #3-98 RT: 0.04-1.34 AV: 96 NL: 5.45E6
T: FTMS + p ESI Full ms [300.00-2000.00]
Relative Abundance
m/z
793.93 z=4
635.35 z=5
753.42 z=4
1058.24 z=3
CRA-4110540_CRA_TG_3025 #3-98 RT: 0.04-1.34 AV: 96 NL: 1.19E6
T: FTMS + p ESI Full ms [300.00-2000.00]
1057.91 z=3
1058.24 z=3
1058.58 z=3
1058.91 z=3
1059.24 z=3
1059.58 z=3
CRA-4110540_CRA_TG_3025 #3-98 RT: 0.04-1.34 AV: 96 NL: 5.45E6
T: FTMS + p ESI Full ms [300.00-2000.00]
793.68 z=4
793.93 z=4
794.18 z=4
794.43 z=4
794.69 z=4
794.94 z=4

CRA_TG 3026 (5)

CRA-4110540_CRA_TG_3026_210430115728 4/30/2021 11:58:56 AM CRA_TG_3026_ITMS

Dissolved in 200ul 50%ACN+2 drops of 0.3%FA, ITMS

CRA-4110540_CRA_TG_3026_210430115728 #25-583 RT: 0.07-1.59 AV: 559 NL: 3.36E5

T: ITMS + p ESI Full ms [300.00-2000.00]

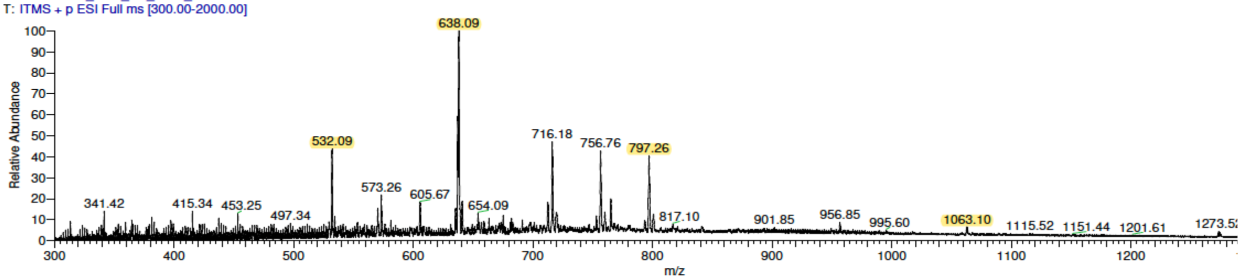

C141H221N37O47 +H: C141 H226 N37 O47 pa Chrg 5

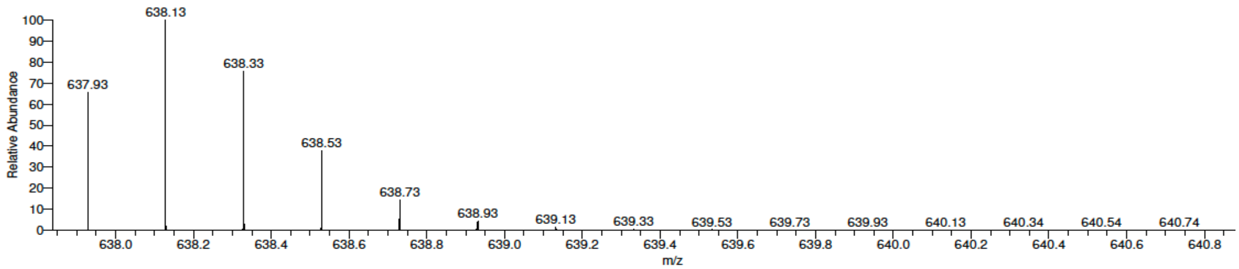

C141H221N37O47 +H: C141 H227 N37 O47 pa Chrg 6

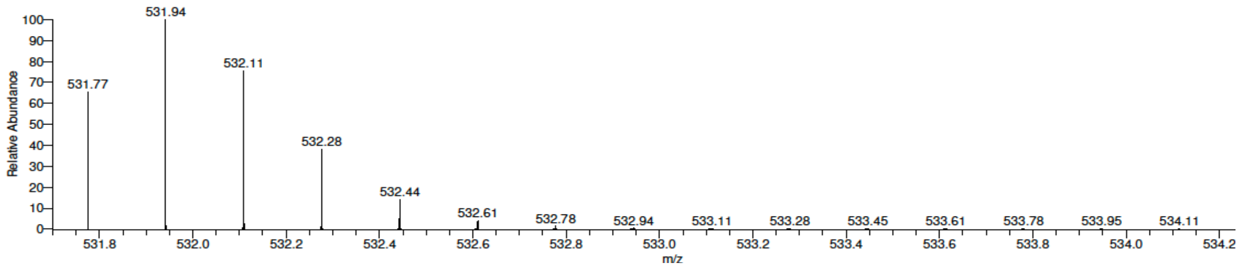

CRA_TG 3027 (6)

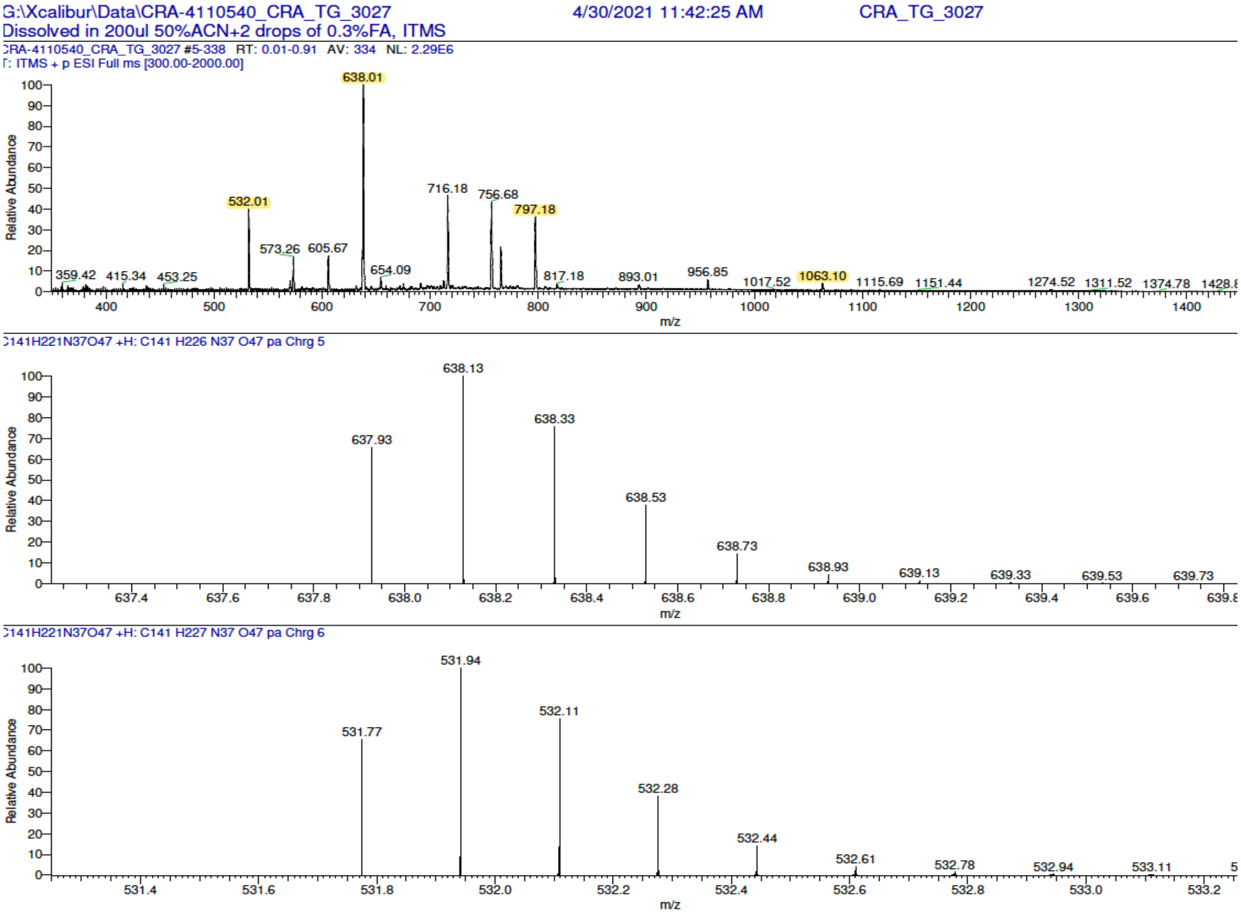

G:\Xcalibur\Data\CRA-4110540_CRA_TG_3027
4/30/2021 11:42:25 AM
CRA_TG_3027
Dissolved in 200ul 50%ACN+2 drops of 0.3%FA, ITMS
CRA-4110540_CRA_TG_3027 #5-338 RT: 0.01-0.91 AV: 334 NL: 2.29E6
T: ITMS + p ESI Full ms [300.00-2000.00]
638.01
532.01
716.18
756.68
797.18
573.26
605.67
654.09
359.42
415.34
453.25
817.18
893.01
956.85
1017.52
1063.10
1115.69
1151.44
1274.52
1311.52
1374.78
Relative Abundance
m/z
C141H221N37O47 +H: C141 H226 N37 O47 pa Chrg 5
638.13
637.93
638.33
638.53
638.73
638.93
639.13
639.33
639.53
639.73
C141H221N37O47 +H: C141 H227 N37 O47 pa Chrg 6
531.94
531.77
532.11
532.28
532.44
532.61
532.78
532.94
533.11

In Vitro cAMP Accumulation

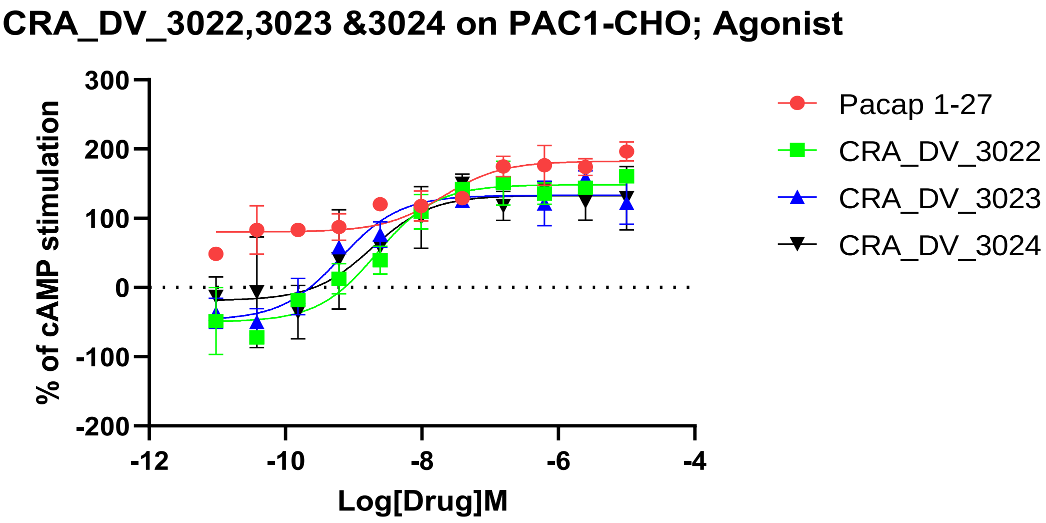

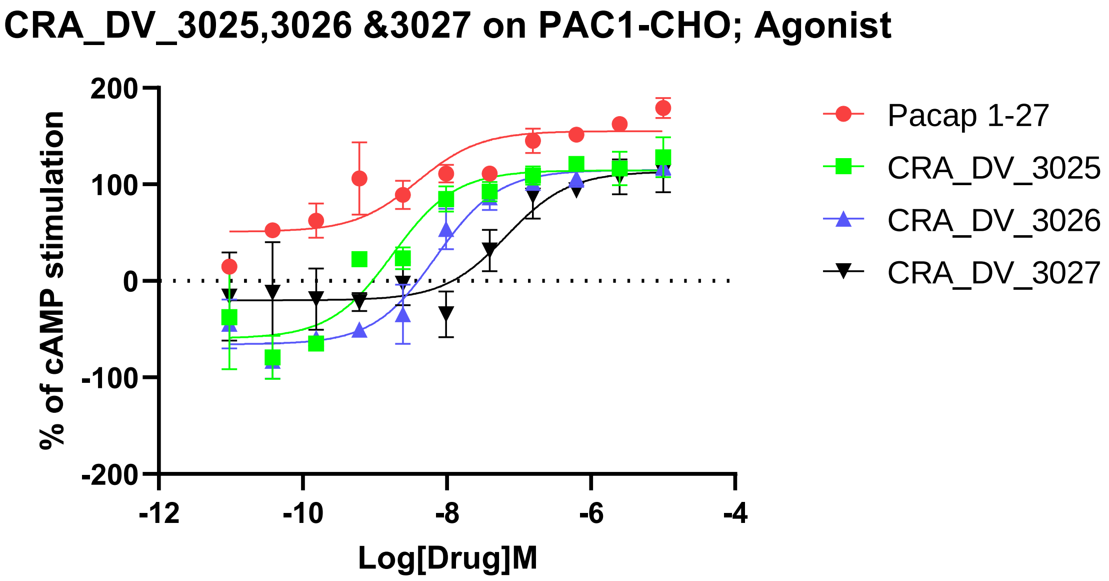

PAC1-CHO	Pacap1-27	CRA_DV_3022	CRA_DV_3023	CRA_DV_3024	VIPR1-CHO	Pacap1-27	CRA_DV_3025	CRA_DV_3026	CRA_DV_3027
**EC50(nM)**	20	2.1	0.6	1.8	**EC50(nM)**	8.9	1.76	6.9	67
**Emax(%)**	100	68	53	52.65	**Emax(%)**	100	57.6	58	56.4
**R^2**	0.82	0.94	0.92	0.78	**R^2**	0.75	0.90	0.96	0.84

Cells: HA-PAC1-CHO Passage #31, (20.000/well) were plated in 3 ea. 96-well plate in DMEM:F12 (1:1)medium, incubated

24 hrs. Cells were serum-starved 4 hrs, then incubated with 500uM IBMX in Serum free media for 20 min. Then treated the cells with serial drug dilutions in vehicle (500uM IBMX in SF media) for 10 minutes incubation. Serial dilutions of different ligands started at 10 uM final concentration. The mixtures were then damped, and ice-cold assay buffer was added, plate was heated at 90 °C for 10 min. Plate was centrifuged at 4000 rpm for 10 min. at 4°C , then supernatant was transferred into a new 96-well plate. Supernatants were co-incubated
with ~ 1 pmol of [3H]cAMP, 7 ug PKA in 0.05% BSA for 1 hr at RT. Data represent mean +/- SEM of a single experiment, carried out in duplicate.

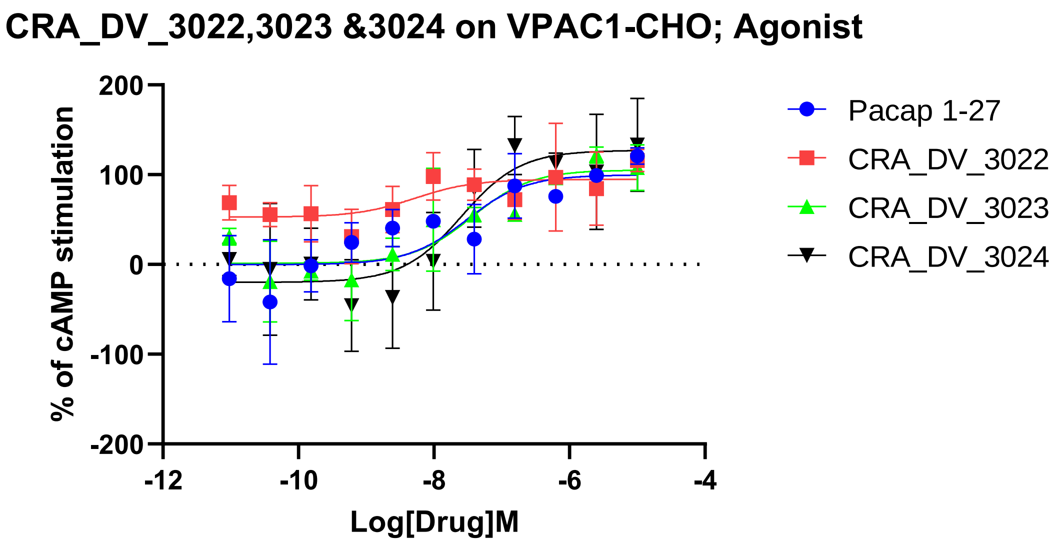

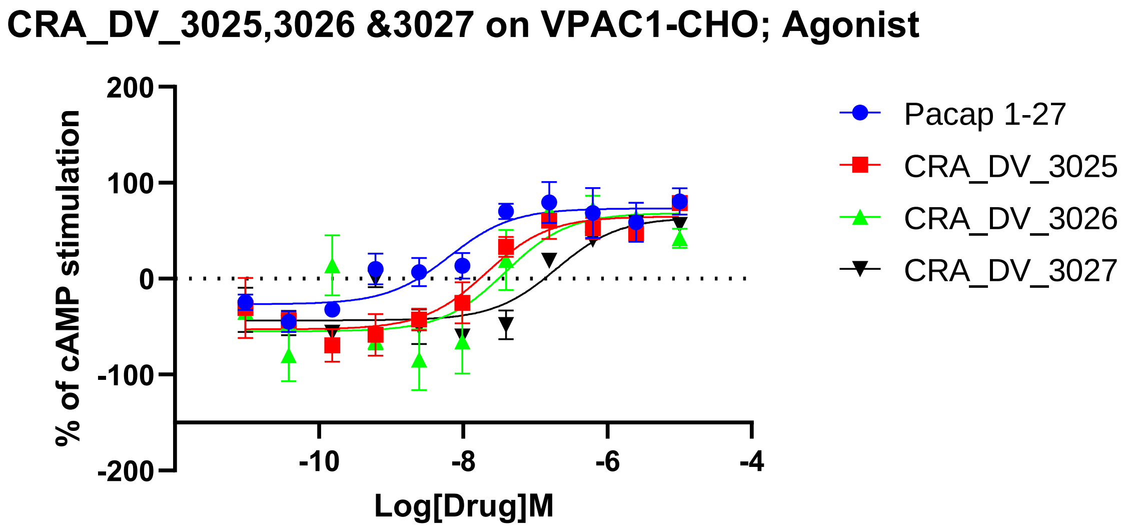

VPAC1-CHO	Pacap1-27	CRA_DV_3022	CRA_DV_3023	CRA_DV_3024	VPAC1-CHO	Pacap1-27	CRA_DV_3025	CRA_DV_3026	CRA_DV_3027
**EC50(nM)**	30	5.2	36	25	**EC50(nM)**	6.4	20	38	195
**Emax(%)**	100	94	105	127	**Emax(%)**	100	91.28	95.21	90.48
**R^2**	0.62	0.32	0.72	0.70	**R^2**	0.85	0.89	0.69	0.81

Cells: HA-VPAC1-CHO Passage #22, (20.000/well) were plated in 3 ea. 96-well plate in DMEM:F12 (1:1)medium, incubated
24 hrs. Cells were serum-starved 4 hrs, then incubated with 500uM IBMX in Serum free media for 20 min. Then treated the cells with serial drug dilutions in vehicle (500uM IBMX in SF media) for 10 minutes incubation. Serial dilutions of different ligands started at 10 uM final concentration. The mixtures were then damped, and ice-cold assay buffer was added, plate was heated at 90 °C for 10 min. Plate was centrifuged at 4000 rpm for 10 min. at 4°C , then supernatant was transferred into a new 96-well plate. Supernatants were co-incubated
with ~ 1 pmol of [3H]cAMP, 7 ug PKA in 0.05% BSA for 1 hr at RT. Data represent mean +/- SEM of a single experiment, carried out in duplicate.

CRA_DV_3022,3023 &3024 on VPAC2-CHO; Agonist

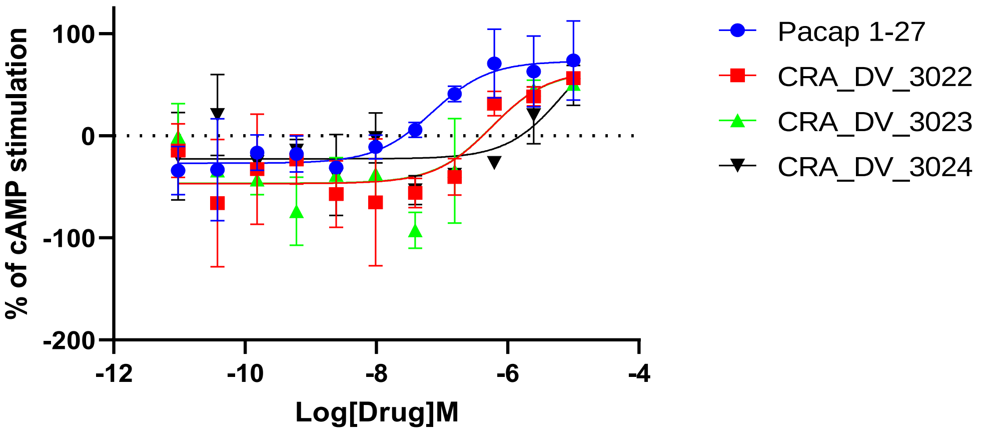

CRA_DV_3025,3026 &3027 on VPAC2-CHO; Agonist

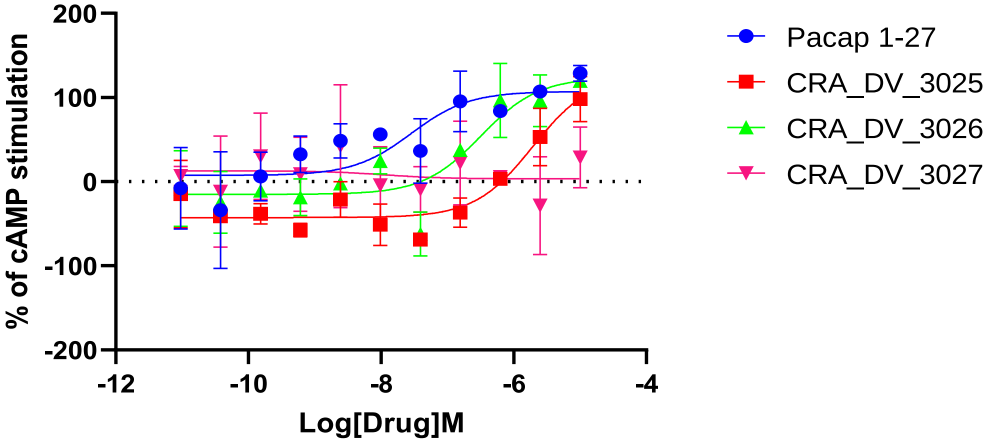

VPAC2-CHO	Pacap1-27	CRA_DV_3022	CRA_DV_3023	CRA_DV_3024	VPAC2-CHO	Pacap1-27	CRA_DV_3025	CRA_DV_3026	CRA_DV_3027
**EC50(nM)**	71	576	577	7369	**EC50(nM)**	28	1943	320	NC
**Emax(%)**	100	91	90.42	130	**Emax(%)**	100	118	115	NC
**R^2**	0.80	0.59	0.61	0.40	**R^2**	0.62	0.82	0.74	NC

Cells: HA-VPAC2-CHO Passage #28, (20.000/well) were plated in 3 ea. 96-well plate in DMEM:F12 (1:1)medium, incubated 24 hrs. Cells were serum-starved 4 hrs, then incubated with 500uM IBMX in Serum free media for 20 min. Then treated the cells with serial drug dilutions in vehicle (500uM IBMX in SF media) for 10 minutes incubation. Serial dilutions of different ligands started at 10 uM final concentration. The mixtures were then damped, and ice-cold assay buffer was added, plate was heated at 90 °C for 10 min. Plate was centrifuged at 4000 rpm for 10 min. at 4°C , then supernatant were transferred into a new 96-well plate. Supernatants were co-incubated with ~ 1 pmol of [3H]cAMP, 7 ug PKA in 0.05% BSA for 1 hr at RT. Data represent mean +/- SEM of a single experiment, carried out in duplicate.
